# Conductive Photo-Activated Porphyrin-ZnO Nanostructured Gas Sensor Array

**DOI:** 10.3390/s17040747

**Published:** 2017-04-01

**Authors:** Gabriele Magna, Alexandro Catini, Raj Kumar, Massimo Palmacci, Eugenio Martinelli, Roberto Paolesse, Corrado di Natale

**Affiliations:** 1 Department of Electronic Engineering, University of Rome Tor Vergata, Via Politecnico 1, 00133 Roma, Italy; magna.gabriele@gmail.com (G.M.); catini@ing.uniroma2.it (A.C.); rajkumar.lmd@gmail.com (R.K.); palmacci@ing.uniroma2.it (M.P.); martinelli@ing.uniroma2.it (E.M.); 2 Department of Chemical Science and Technology, University of Rome Tor Vergata, Via della Ricerca Scientifica, 00133 Roma, Italy; roberto.paolesse@uniroma2.it

**Keywords:** gas sensor arrays, porphyrins, ZnO, nanoparticles

## Abstract

Chemoresistors working at room temperature are attractive for low-consumption integrated sensors. Previous studies show that this feature can be obtained with photoconductive porphyrins-coated ZnO nanostructures. Furthermore, variations of the porphyrin molecular structure alter both the chemical sensitivity and the photoconductivity, and can be used to define the sensor characteristics. Based on these assumptions, we investigated the properties of an array of four sensors made of a layer of ZnO nanoparticles coated with porphyrins with the same molecular framework but different metal atoms. The array was tested with five volatile organic compounds (VOCs), each measured at different concentrations. Results confirm that the features of individual porphyrins influence the sensor behavior, and the differences among sensors are enough to enable the discrimination of volatile compounds disregarding their concentration.

## 1. Introduction

Organically capped ZnO nanostructures have been widely studied—in particular for their optical and electronic properties [1]. A noteworthy example is provided by the combination of porphyrins and ZnO. In most cases, the performance of these hybrid materials may exceed that of the individual constituents. For instance, the photo-activated charge transfer from porphyrin to ZnO has been found to lead to an enhancement of photovoltaic properties [2] and catalytic efficiency [3,4].

Since porphyrins are extremely suitable as a chemically sensitive material [5], we have been interested in investigating the sensing qualities of porphyrins-coated ZnO nanostructures. We found that in porphyrins-coated nanorods, the exposure to visible light largely improved the sensitivity of the conductivity with respect to electron donating species such as amines [6]. Further studies have shown that a similar behavior is also shown by the surface potential measured by a Kelvin probe [7].

An important issue in these materials is the functionalization process. The standard method used to achieve organically-capped nanostructures consists of the deposition of an organic layer onto the surface of pre-formed inorganic structures. However, alternative routes where a more deep interaction between organic an inorganic molecules can be conceived. In this regard, we found that the addition of porphyrins to the ZnO hydrothermal growth solution largely alters the nanostructures order, but on the other hand, it results in a compact and thin organic coating. Furthermore, the one-pot growth confers peculiar properties to sensors which are different from those made with the standard fabrication route [8,9].

In spite of these promising results, the effective exploitation of these sensors is made difficult by the geometry of nanorods. Hydrothermal growth proceeds from a seed layer which forms—after the synthesis—a sort of basement of the vertical rods. When performed on planar electrodes (as usually happens in thin film processing), this assemblage results in a lack of influence of the nanorods on the observed conductivity. In other words, the vertical nanorods are almost completely shortened by the seed layer basement. This problem can be avoided by patterning the seed layer as the two electrodes. In this way, the hydrothermal growth gives rise to nanorods connecting one electrode to the other. This approach has been shown to be viable [10], but too laborious for a simple and low-cost sensor manufacturing method.

Nanoparticles are a straightforward alternative. They can be grown in solution and deposited as a solid film onto the sensor substrate with a simple dip coating process. Furthermore, in contrast to nanorods, the homogeneous surface of nanoparticles facilitates the coating process.

In this paper, we investigate the sensor properties of an array of four ZnO nanoparticles, each coated with a different porphyrin. The functionalized nanoparticles were fabricated following the one-pot method introduced for nanorods. Porphyrins-coated nanoparticles were deposited onto a substrate endowed with interdigitated electrodes for conductivity measurement. The sensor array was tested by measuring the sensitivity towards five volatile compounds, each representative of a chemical family of compounds: organic acid, alcohol, arene, amine, and water. Due to its chemical peculiarity, each compound is expected to interact with porphyrins with a different blend of fundamental interactions, including van der Waals forces, hydrogen bonds, π–π interactions, and coordination. Results show that the array of porphyrins can identify the five compounds, even if they are presented at different concentration. This suggests that porphyrins-coated nanoparticles are a viable material for the development of resistive gas sensors.

## 2. Experimental Section

5-(4’-Carboxyphenyl)-10,15,20-triphenylporphyrinato (H_2_TPPCOOH) and its metal complexes with copper (CuTPPCOOH), cobalt(II) (CoTPPCOOH), and zinc (ZnTPPCOOH) have been prepared following literature methods [11].

**Figure 1:**
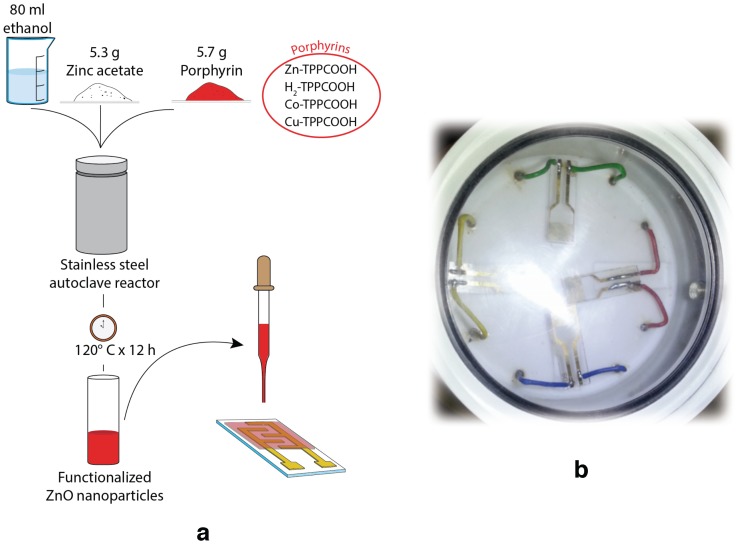
(**a**) Steps of the sensor preparation procedure; (**b**) Picture of the sensors cell with four sensors. H_2_TPPCOOH: 5-(4’-carboxyphenyl)-10,15,20-triphenylporphyrinato; CoTPPCOOH: H_2_TPPCOOH metal complex with cobalt(II); CuTPPCOOH: H_2_TPPCOOH metal complex with copper; ZnTPPCOOH: H_2_TPPCOOH metal complex with zinc.

Porphyrins-coated ZnO nanoparticles were prepared with a one-pot procedure. The method follows the procedure optimized for nanorods coating [8], where porphyrins are added to the ZnO precursor solution [12]. For the scope, 5.7 mg of porphyrins were added to 5.3 g of zinc acetate added into 80 mL of ethanol. A Teflon cylinder was filled with the solution and sealed in a stainless autoclave. The synthesis occurred at 120 °C for 12 h. Impurities were removed by washing the precipitate with ethanol. Figure 1a shows the sensor preparation steps. Nanoparticles were morphologically characterized by transmission electron microscopy (TEM) (FEI Tecnai G2 Spirit, Hillsboro, OR, USA).

**Figure 2:**
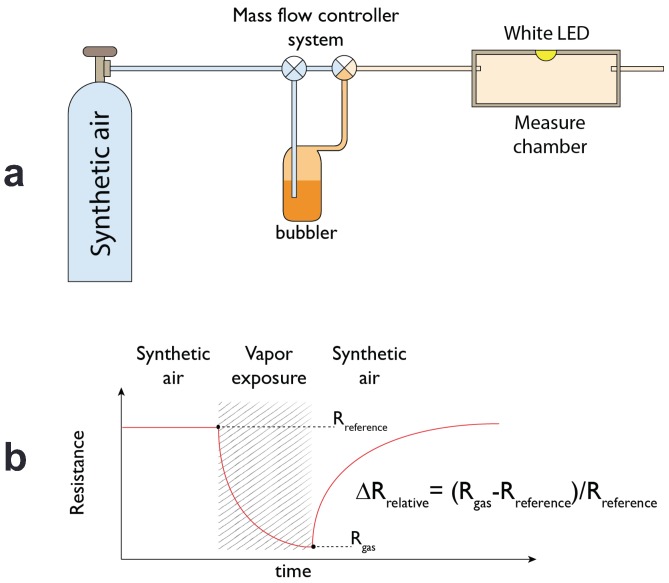
(**a**) Scheme of the measurement setup; (**b**) The sensor response is calculated as the relative change of resistance measured in reference and during the addition of volatile compound.

Solid films of porphyrins-coated ZnO nanoparticles were made by drop coating technique onto a glass substrate interdigitated electrodes (DropSense), visible in Figure 1b. Sensors were placed in a sealed cell with a transparent optical window (see Figure 1b). Sensors were tested with vapors of five volatile compounds: acetic acid, pentanol, toluene, water, and triethylamine. The liquids were kept at the constant temperature of 303 K. The saturation pressure was diluted with a carrier of synthetic air at different dilution percentages from 2% to 15% by mass-flow controllers (from MKS Instruments, Andover, MA, USA) that maintained the total flux at 200 cm^3^/min. Sensors were connected to a multimeter (Keithley Instruments, Solon, OH, USA) that sequentially measured the resistance of the four sensors. During the measurement, sensors were illuminated with a white LED (1 W High Power LED Light Source, ASMT-AW00, Broadcom Limited, San Jose, CA, USA). The LED emission spectrum fits with the absorbance spectra of porphyrins [6]. The relative change of resistance—measured before and at the end of the exposure—was used as the sensor response. Figure 2 shows the measurement setup and the sensor signal definition.

## 3. Results and Discussion

**Figure 3:**
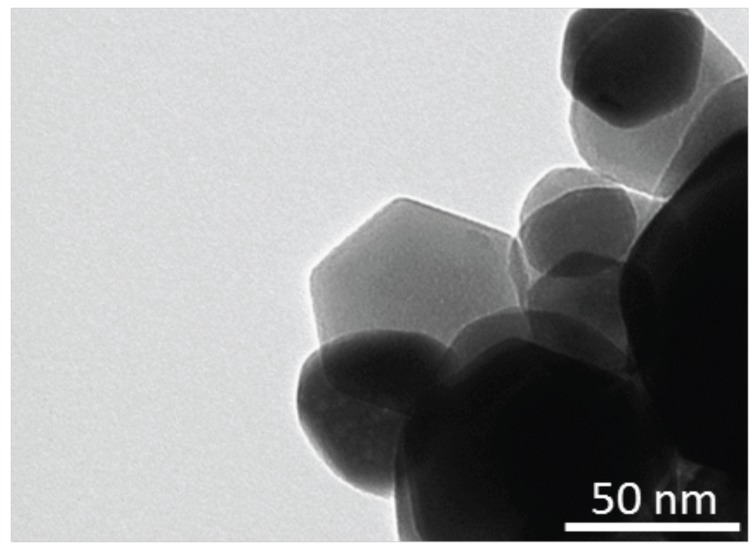
TEM picture of porphyrins-coated ZnO nanoparticles.

Figure 3 shows a TEM picture of porphyrins-coated ZnO nanoparticles. As previously observed with nanorods [9], the addition of porphyrins to the ZnO precursor solution strongly affects the final morphology of the nanostructure. The particles diameter is on the order of 50 nm, which is smaller than that obtained without porphyrins [13]; furthermore, the particles are characterized by rounded borders and irregular shapes.

**Figure 4:**
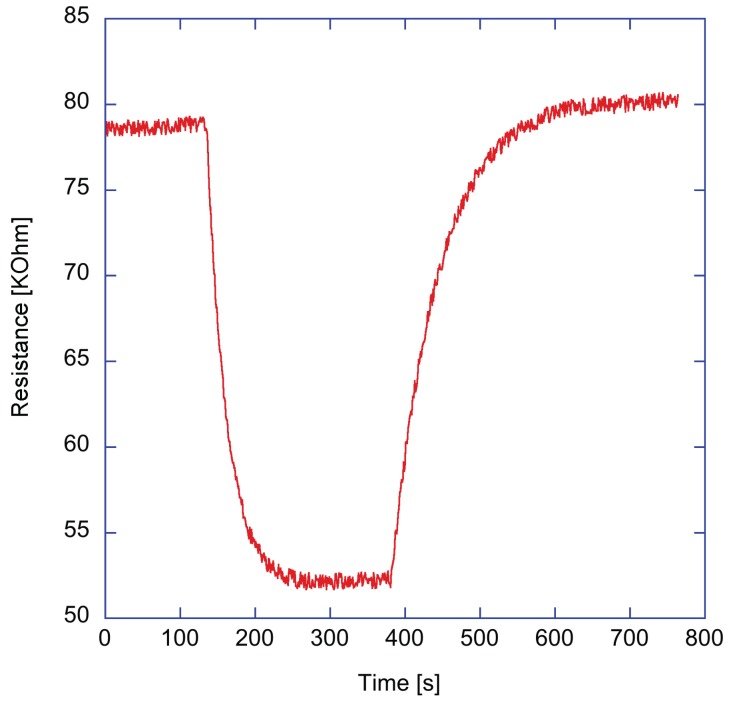
Evolution of the resistance of ZnTPPCOOH-coated ZnO nanoparticles layer exposed to 2% of saturated pressure of triethylamine.

During the gas measurements, the sensors were always kept under the constant illumination of the white LED. The resistances in this condition were in the range of tens of KΩ. As an example, Figure 4 shows the resistance change of ZnTPPCOOH sensor exposed to 2% of triethylamine vapors. A good recovery of the baseline can be observed. As shown in Figure 2b, the sensor response is the ratio between the resistance change and the baseline value.

**Figure 5:**
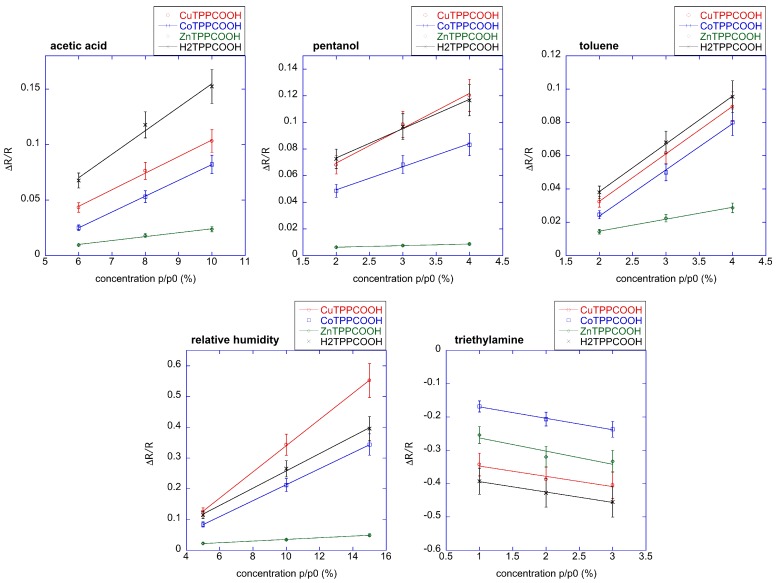
Response curve of the four sensors to the five volatile compounds. Concentration interval is between 1% of saturated vapor for triethylamine up to 15% of saturated vapour in the case of water. The linear fit is also plotted.

Figure 5 shows the response curves of the four sensors to the five volatile compounds. Since each volatile compound is characterized by a different saturation pressure, the concentrations in the gaseous phase are different. The saturation pressures are in the interval between 0.6 KPa (pentanol) up to 5.95 KPa (triethylamine). The ratio p/p0 is the relative concentration, and in case of water vapour it is the relative humidity. Measures have been repeated in triplicate, and the dispersion of the signals is shown in Figure 4. The dispersion was less than 10%.

As found in other porphyrins-based sensor arrays, all sensors show a common behavior. This evidences the fact that the chemical difference between porphyrins—here limited only to the metal ion complexed at the core of the macrocycle—is a small factor with respect to the influence of the whole macrocycle.

In all sensors, the absorption of triethylamine elicits a decrease of conductivity in all sensors, but the resistance increases for all the other volatile organic compounds (VOCs).

The peculiar response to triethylamine may be explained considering that amines are electron donors and bases. The coordination of the amine explains the behavior of metalloporphyrins (CuTPPCOOH, CoTPPCOOH, and ZnTPPCOOH). However, the lack of metal makes the coordination with the free-base porphyrin (H_2_TPPCOOH) impossible. In this case, the sensitivity can be justified considering that the core of the free-base porphyrins shows an amphoteric character, the imine group (-C=N-) being basic and the pyrrole-type nitrogen atoms (N-H) acid. Then, instead of the coordination to the metal ion, the sensitivity of H_2_TPPCOOH with respect to triethylamine is likely due to an acid–base interaction.

Finally, it is important to remark on the role of the illumination, which promotes the electron transfer from the porphyrin to the ZnO, leaving the porphyrin depleted of electrons and then more prone to accept electrons from the donating triethylamine. Thanks to these mechanisms, both the coordination and the acid–base interaction give rise to an increase of the conductivity of the sensor.

The interaction with the other volatile compounds increases the resistance. The reasons for this behavior are not completely clear; the observed decrease of conductivity is likely contributed by a reduced efficiency of the photocurrent that could be due to the fact that a quantity of photoexcited electrons are engaged in the interaction with the volatile compound. More studies are necessary to clarify these mechanisms. Negative and positive changes of conductivity were also shown by ZnTPPCOOH-coated ZnO nanrods with respect to triethylamine and ethanol [6].

In the explored concentration range, the response curves are linear. In this regard, it is worth noting that the interval of concentration is rather narrow, being 2% for pentanol, toluene, and triethylamine, 4% for acetic acid, and only in the case of water, 10% of interval relative humidity. Consequently, the linearity may also be due to the local linearization of a more general non-linear isotherm.

**Figure 6:**
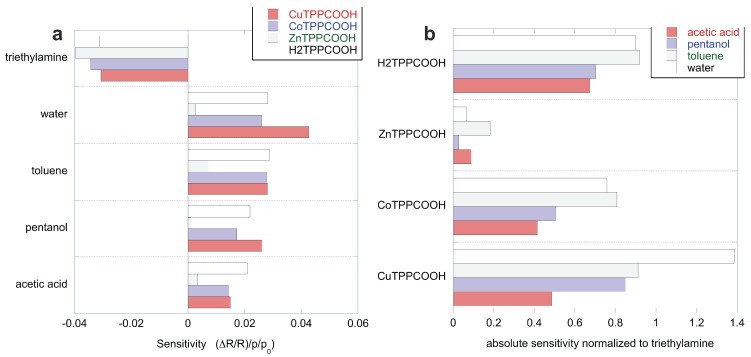
(**a**) Sensitivity of each sensor to the five volatile compounds; sensitivities are calculated in the investigated concentration range; (**b**) Same data of Figure 6a but normalized respect to the sensitivity to triethylamine.

The sensitivity of the sensors to the five compounds is calculated as the derivative of the sensor response (ΔR/R) with respect to the concentration (p/p0). All the sensitivities are compared in Figure 6a. As discussed above, the sensors are characterized by a common behavior with respect to the set of VOCs. Since the sensitivity to triethylamine is peculiar, it is interesting to evaluate the sensitivity to the other VOCs with respect to triethylamine. The ratio of sensitivity accounts for the selectivity of the sensor with respect to triethylamine. Figure 6b shows the sensitivity normalized to the absolute value of the sensitivity to triethylamine. Except for the case of the sensitivity of CuTPPCOOH to water, the ratio of sensitivities is always smaller than one. The smallest normalized sensitivities are found in ZnTPPCOOH, which is the more selective of the four sensors. It is important to observe that each sensor shows a different pattern of relative sensitivity. This feature is particularly important for sensor arrays, because the sensor’s capability to discriminate compounds relies on these differences.

Principal component analysis (PCA) was used to investigate the collective behavior of the four sensors. For the scope, the sensor responses were arranged in a matrix, and PCA was calculated on the autoscaled (zero mean and unitary variance) matrix [14].

PCA was applied to the whole set of measurements, where each concentration was measured three times. In this way, PCA results enable an appreciation of the separation of the different VOCs with respect to the dispersion of sensor signals.

**Figure 7:**
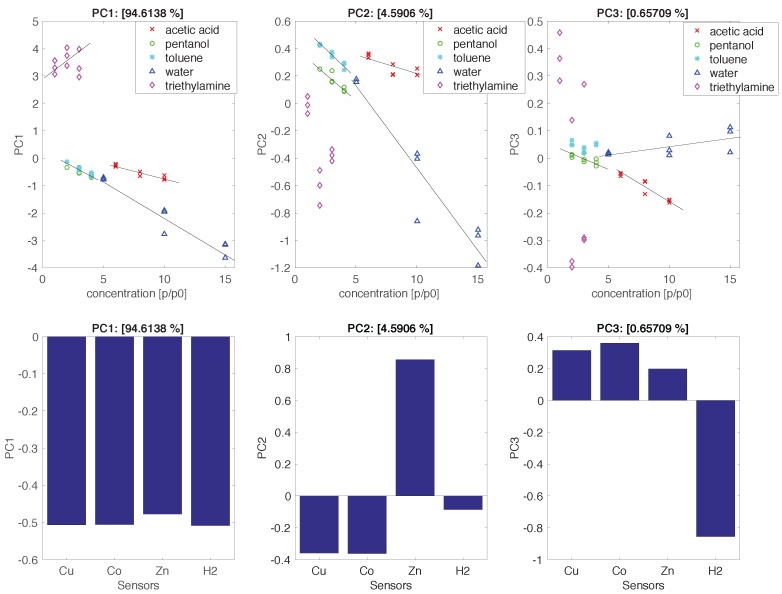
Scores and loadings of the first three principal components. Scores (upper) are plotted versus concentration, while loadings are plotted as a bar plot.

Figure 7 shows the scores and the loadings of the first three principal components of the autoscaled data matrix. The variance explained in each principal component is indicated in the title of each plot in Figure 7. As found with correlated sensors, the first principal component carries a large part of the total variance (94%), and all the sensors almost equally contributed to PC1.

Two main sources of correlation can be found in our data: the intrinsic correlation among the sensors (evidenced by the similarities of the sensitivity patterns in Figure 6), and the correlation due to the properties of the samples. In this experiment, the last contribution is attributable to the varying concentration of compounds. Indeed, since sensor responses are proportional to the concentration (see Figure 5), the variable concentration introduces a strong correlation in the data. The influence of concentration is clearly visible in the first principal component, which is strongly correlated to the concentration regardless of the kind of compound. The correlation of the scores with the concentration is attenuated in the second and third principal components. For these components, the contribution of the sensors is also rather sparse.

The individual character of each sensor—which is not very evident from sensitivities comparison—can be easily appreciated considering the principal components. For instance, the second principal component shows a divergence between ZnTPPCOOH and the other sensors. This difference is expected, considering that ZnTPPCOOH is the more selective sensor of the array. On the other hand, the chemical disparity between metalloporphyrins and free-base porphyrin is captured by the third principal component.

The optimal identification of VOCs requires the removal of the influence of the concentration on the sensor responses. This would limit the sensor response to only the qualitative character of the measured compounds.

The segregation of qualitative from quantitative information has been a well-known problem since the beginning of studies of sensor arrays. In the case of linear sensors, this can be achieved by a simple normalization of the sensor response [15]. This consists of dividing the response of each sensor by the sum of the response of all the sensors of the array. In the case of an array of N linear sensors, the response of the *i*-th sensor ($\Delta R_{ij}/R_{i}$) to the *j*-th compound at concentration c ($c_{j}$) is:

(1) $$\frac{\Delta R_{ij}}{R_{i}}=S_{ij}\cdot c_{j}$$

where $S_{ij}$ is the sensitivity of the *i*-th sensor to the *j*-th compound.

The normalized sensor response, ${\left(\Delta R/R\right)}^{*}$, is calculated with the following equation:

(2) $${\left(\frac{\Delta R_{ij}}{R_{i}}\right)}^{*}=\frac{\frac{\Delta R_{ij}}{R_{i}}}{\sum_{k}\frac{\Delta R_{kj}}{R_{k}}}=\frac{S_{ij}c_{j}}{\sum_{k}S_{kj}c_{j}}=\frac{S_{ij}}{\sum_{k}S_{kj}}$$

In practice, the signal of the *i*-th sensor corresponds to the weighted sensitivity.

**Figure 8:**
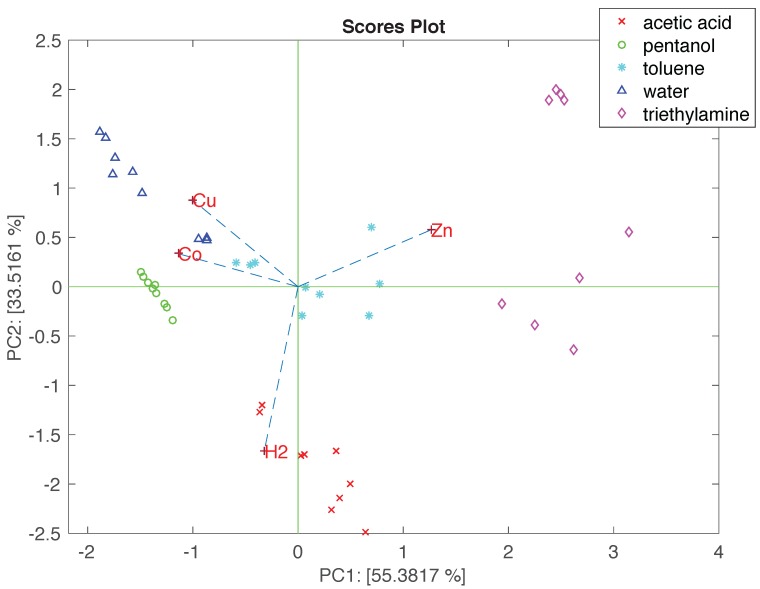
Bi-plot of the first two principal components of the matrix of linearly normalized data. Loadings are labelled with the first two letters of the corresponding porphyrin.

PCA was applied to the matrix of normalized data. Figure 8 shows the PCA results as a bi-plot where scores and loadings are contemporaneously displayed. Such a plot allows for studying—in addition to the clustering—the relationship between the data and the sensors. In Figure 8, the data related to each compound cluster together, disregarding the concentration. Due to the peculiar sensors responses, triethylamine is plotted aside. The loadings of ZnTPPCOOH—the most selective sensor—points towards the region where triethylamine is plotted. The other compounds are more gathered together, but without overlaps. CoTPPCOOH and CuTPPCOOH point toward pentanol and water. This is not surprising, considering that CuTPPCOOH shows the largest sensitivity to these compounds. Finally, H_2_TPPCOOH is oriented towards the acetic acid, in agreement with the fact that this is the sensor with the largest sensitivity to acetic acid.

The PCA of normalized data is consistent with the sensitivity pattern, and it shows that these sensors—even if rather similar to one another—are also significantly different to enable the discrimination of the tested compounds.

## 4. Conclusions

Porphyrins-based gas sensor arrays have been shown to be effective in many different fields, from food analysis to medical diagnosis [5]. These sensors have been almost exclusively based on either mass or optical transducers. However, from the electronic point of view, the implementation and integration of gas-sensitive resistors is the most straightforward. An important advance in the exploitation of the sensing properties of porphyrin is then expected if they could be matched with resistive transducers. The combination of porphryins with a conductive material is a practical approach for resistive porphyrin-based sensors.

This paper investigated this concept, studying the behavior of sensors made of ZnO nanoparticles coated with three metalloporphyrins, made of cobalt, copper, and zinc, and the respective free-base porphyrin. All these sensors show a peculiar sensitivity to triethylamine (a strong electron donor), but also a different pattern of interactions with the other tested volatile compounds. These differences appear subtle when sensors are individually compared, but are sufficiently large to identify different volatile compounds regardless of the variation of concentration.

This paper was focused on the demonstration that a chemo-resistive porphyrins-based sensor array can be obtained by exploiting the photo-current generated by the porphyrins—which are typically non-conductive— when they coat the surface of ZnO. However, it remains to be demonstrated that the change of resistance can efficiently transduce all the interactions that can take place between the porphyrins and the volatile compounds and that such an array can actually be used to discriminate among real samples.
